# Non-Invasive Prenatal Diagnosis of Monogenic Disorders Through Bayesian- and Haplotype-Based Prediction of Fetal Genotype

**DOI:** 10.3389/fgene.2022.911369

**Published:** 2022-07-01

**Authors:** Jia Li, Jiaqi Lu, Fengxia Su, Jiexia Yang, Jia Ju, Yu Lin, Jinjin Xu, Yiming Qi, Yaping Hou, Jing Wu, Wei He, Zhengtao Yang, Yujing Wu, Zhuangyuan Tang, Yingping Huang, Guohong Zhang, Ying Yang, Zhou Long, Xiaofang Cheng, Ping Liu, Jun Xia, Yanyan Zhang, Yicong Wang, Fang Chen, Jianguo Zhang, Lijian Zhao, Xin Jin, Ya Gao, Aihua Yin

**Affiliations:** ^1^ BGI Genomics, BGI-Shenzhen, Shenzhen, China; ^2^ Hebei Industrial Technology Research Institute of Genomics in Maternal and Child Health, Shijiazhuang BGI Genomics, Shijiazhuang, China; ^3^ Medical Genetics Centre, Guangdong Women and Children’s Hospital, Guangzhou Medical University, Guangzhou, China; ^4^ BGI-Shenzhen, Shenzhen, China; ^5^ Shenzhen Engineering Laboratory for Birth Defects Screening, Shenzhen, China; ^6^ Prenatal Diagnosis Centre, Guangdong Women and Children’s Hospital, Guangzhou, China; ^7^ Maternal and Children Metabolic-Genetic Key Laboratory, Guangdong Women and Children’s Hospital, Guangzhou, China; ^8^ College of Life Sciences, University of the Chinese Academy of Sciences, Beijing, China; ^9^ College of Medical Technology, Hebei Medical University, Shijiazhuang, China

**Keywords:** non-invasive prenatal diagnosis, massively parallel sequencing, fetal genome, single nucleotide variations, monogenic disease

## Abstract

**Background:** Non-invasive prenatal diagnosis (NIPD) can identify monogenic diseases early during pregnancy with negligible risk to fetus or mother, but the haplotyping methods involved sometimes cannot infer parental inheritance at heterozygous maternal or paternal loci or at loci for which haplotype or genome phasing data are missing. This study was performed to establish a method that can effectively recover the whole fetal genome using maternal plasma cell-free DNA (cfDNA) and parental genomic DNA sequencing data, and validate the method’s effectiveness in noninvasively detecting single nucleotide variations (SNVs), insertions and deletions (indels).

**Methods:** A Bayesian model was developed to determine fetal genotypes using the plasma cfDNA and parental genomic DNA from five couples of healthy pregnancy. The Bayesian model was further integrated with a haplotype-based method to improve the inference accuracy of fetal genome and prediction outcomes of fetal genotypes. Five pregnancies with high risks of monogenic diseases were used to validate the effectiveness of this haplotype-assisted Bayesian approach for noninvasively detecting indels and pathogenic SNVs in fetus.

**Results:** Analysis of healthy fetuses led to the following accuracies of prediction: maternal homozygous and paternal heterozygous loci, 96.2 ± 5.8%; maternal heterozygous and paternal homozygous loci, 96.2 ± 1.4%; and maternal heterozygous and paternal heterozygous loci, 87.2 ± 4.7%. The respective accuracies of predicting insertions and deletions at these types of loci were 94.6 ± 1.9%, 80.2 ± 4.3%, and 79.3 ± 3.3%. This approach detected pathogenic single nucleotide variations and deletions with an accuracy of 87.5% in five fetuses with monogenic diseases.

**Conclusions:** This approach was more accurate than methods based only on Bayesian inference. Our method may pave the way to accurate and reliable NIPD.

## Introduction

Following the discovery of fetal cell-free DNA (cfDNA) in maternal plasma (Lo et al., 1997), next-generation sequencing technologies have enabled non-invasive prenatal screening for trisomies 13, 18, and 21; aneuploidies involving sex chromosomes (Chen et al., 2011; Agarwal et al., 2013; Benn et al., 2013; Mazloom et al., 2013; Samango-Sprouse et al., 2013; Hooks et al., 2014); and, more recently, rare autosomal aneuploidies and various sub-chromosomal aberrations (Snyder et al., 2015; Zhou et al., 2019). Maternal plasma cfDNA testing is now being applied to non-invasive prenatal diagnosis (NIPD) of monogenic diseases. So far, such testing has involved whole-exome sequencing of the cfDNA and analysis supplemented by parental haplotype information (Fan et al., 2012a; Lam et al., 2012; Yoo et al., 2015; Zhang et al., 2015). However, these methods can detect only autosomal dominant diseases and a few autosomal recessive diseases caused by known mutations. They cannot detect diseases that have not already been associated with mutation hotspots or that are caused by *de novo* variants (DNVs).

Another limitation of these methods is that they require proband genomic DNA to allow haplotype phasing, which may not be feasible if the proband passes away at the early life. To avoid this requirement, two groups developed methods to infer fetal genotype based on haplotyping of one or both parents. One method was able to detect only ∼66%–70% of paternal-specific alleles and deduce only ∼70% of paternally inherited haplotypes (Fan et al., 2012b), while the other method predicted heterozygous maternal and homozygous paternal (ABAA) loci with only 64.4% accuracy, and it was unable to predict many heterozygous maternal and paternal (ABAB) loci for lack of paternal haplotype information (Kitzman et al., 2012). Moreover, these methods cannot detect variants for which no haplotype or genome phasing information is available.

In the present study, we have established a Bayesian model that predicts fetal genotype based on whole-genome sequencing of cfDNA in maternal plasma and of single-tube long fragment reads (stLFRs) in paternal genomic DNA. This allows the reconstruction of parental haplotypes without the need of proband DNA, which in turn renders the fetal genotyping more accurate. We validate the effectiveness of our approach by non-invasively detecting single-nucleotide variants (SNVs), insertions and deletions (InDels) in five fetuses at risk of monogenic diseases.

## Methods and Materials

### Study Design and Study Population

Five mothers with normal singleton pregnancies and their male partners were prospectively recruited into this study at the Department of Fetal Medicine and Prenatal Diagnosis at Guangdong Women and Children’s Hospital. All five pregnant women showed normal nuchal translucency (NT), and non-invasive prenatal screening results were negative for trisomies 13, 18, and 21. All women delivered healthy babies by vaginal delivery (Supplementary Table S1). Peripheral blood (5 ml) of each mother and father was sampled into an ethylenediamine tetraacetic acid-containing tube to provide information for the haplotype- and Bayesian-based method, the results of which were compared against umbilical cord blood (2 ml).

To validate our method for detecting pathogenic SNVs and indels for NIPD, another five mothers and their male partners whose fetuses were at risk of the following monogenic diseases were also prospectively recruited: tetrahydrobiopterin deficiency hyperphenylalafivemia, Duchenne/Becker muscular dystrophy, ocular albinism, muscular dystrophy polysaccharide glycosylation deficiency A11 and deafness. The five families were recruited because the parents were known to be carriers of disease alleles, or the fetuses were suspected of having monogenic diseases due to ultrasound abnormalities. All five pregnant women agreed to undergo amniocentesis for prenatal diagnosis, and maternal and paternal genomic DNA was Sanger-sequenced to confirm the presence of disease variants.

All families received a detailed explanation of the study and gave written informed consent before any samples were collected. The study strictly followed the Declaration of Helsinki and was approved by the Ethics Committee of the Guangdong Women and Children’s Hospital (no. 201901091), as well as by the Institutional Review Board of the BGI (BGI-IRB 20002).

### Preparation of cfDNA Libraries

Maternal blood was collected and within 8 h, it was centrifuged at 1,600 g for 10 min. Plasma was transferred to fresh microcentrifuge tubes and centrifuged at 16,000 g for 10 min to remove residual cells. From 600 μl of the clarified plasma was extracted cfDNA using the MGIEasy Circulating DNA Isolation Kit (MGI, Shenzhen, China), which was used to construct a library with the MGIEasy Cell-free DNA Library Prep Kit (MGI, Shenzhen, China) based on a modified protocol (Xu et al., 2019). In brief, the extracted cfDNA was end-repaired, ligated with “A” tailing and then ligated with adapters. The ligated products were cleaned up and subjected to 10 cycles of PCR amplification. The PCR products were cleaned up, quantitated with a dsDNA Fluorescence Assay Kit (Invitrogen, United States), heat-denatured and incubated at 37°C to create ssDNA circles. These circles were subjected to rolling circle amplification to generate DNA nanoballs (Drmanac et al., 2010).

### Preparation of Parental gDNA Libraries

High-molecular-weight parental genomic DNA was isolated from blood using a dialysis-based method (Wang et al., 2019) and prepared for stLFR sequencing, in which the same barcode sequence was added to subfragments of long DNA molecules to enable their second-generation sequencing (Wang et al., 2019). The resulting high-molecular-weight parental DNA (1.5 ng) was used to construct a library with the MGIEasy stLFR Library Prep Kit (MGI, Shenzhen, China). In brief, a hybridization sequence of 200–1,000 bp was added to the genomic DNA using transposons, and the resulting transposon-integrated DNA was allowed to adsorb onto beads. The transposons were ligated to barcode adapters, followed by other adaptors to allow multiplex sequencing. The ligated products were cleaned, subjected to five cycles of PCR amplification, purified and quantified using the Qubit^®^ dsDNA HS Assay Kit (Invitrogen, United States).

### Preparation for Umbilical Blood DNA Libraries

Umbilical blood DNA was extracted using an MGIEasy Magnetic Beads Genomic DNA Extraction Kit (MGI, Shenzhen, China), then used to prepare a library with the MGIEasy universal DNA Library Prep Set (MGI). Genomic DNA was fragmented with Segmentase (MGI, Shenzhen, China) to generate molecules 100–500 bp long, and fragments 280–320 bp were enriched using magnetic beads. The ends were filled in, then the base A was added to the 3′ end to allow DNA fragments to be ligated to an adapter with base T at the 3′ end. The DNA fragments were amplified by ligation-mediated PCR and purified to form the library.

### Library Quality Control and Sequencing

Library control was checked using an Agilent DNA 1000 kit on a Bioanalyzer 2,100 platform (Agilent, United States) and quantified using a QubitTM ssDNA Assay Kit (Invitrogen, United States). Then libraries were subjected to multiplex sequencing on a DNBSEQ platform (MGI, Shenzhen, China) acording to a “paired-end 100 bp” strategy.

### Read Mapping and Variant Calling

Raw reads were trimmed and filtered using SOAPnuke 2.1.1 (Chen et al., 2018). Reads were excluded if their N proportion was below 0.1, if > 50% of bases had a quality score <12, or if there were >2 mismatches with the adapter. The resulting “clean reads” were aligned to the human genome reference (hg19) using Burrows-Wheeler Aligner (BWAmem) software (Li and Durbin, 2009), then duplicate reads were removed, InDels were realigned, and base quality scores were recalibrated using Sentieon genomics software2 (Kendig et al., 2019) and default parameters. Variants were called using Sentieon genomics software2. SNVs and InDels were detected using Sentieon DNAscope software (McKenna et al., 2010), which combines the GATK’s HaplotypeCaller and a genotyping model based on machine learning.

### Prediction of Fetal Genotype Using a Bayesian Model

FF was calculated by comparing the aligned sequence reads at maternal homozygous sites and fetal heterozygous sites using the formula FF = 2p/(p + q)×100, where p is the number of reads corresponding to fetal-specific alleles and q the number of reads shared between the mother and fetus. A Bayesian model was used to infer fetal genotype at each locus based on the FF and parental genotyping. The Bayesian model proceeded in two steps. First, the cumulative probability of the combination of each maternal and fetal genotype at each locus was calculated based on the read depth and FF at that locus. Second, the prior probabilities of the maternal and fetal genotype combinations were determined based on parental genotyping and Mendelian laws of inheritance. The Bayesian model generated 10 posterior probabilities, one for each possible combination of maternal and fetal genotypes. The predicted fetal genotype at each locus was the genotype with the highest posterior probability (Eq. 1)

$$P\left(A_{i}\text{|}B\right)=\;\frac{P\left(B\text{|}A_{i}\right)P\left(A_{i}\right)}{\sum_{i=1}^{n}P\left(B\text{|}A_{i}\right)P\left(A_{i}\right)}$$ (1)

where P (Ai) is the prior probability of the *i*th maternal and fetal genotype combination, calculated according to Mendelian laws; P (B|Ai) is the cumulative probability of maternal and fetal genotype combination i based on read depth and FF at that locus; and n was 10 maternal and fetal genotype combinations.

For each locus, the probability of obtaining base j was calculated as follows: (Eq. 2):

Pj=BjF/2∗ C+BjM/2∗(1−C) (2)

where BjF, an integer between 0 and 2, indicates the number of bases j in F1iF2i; C represents the FF in the predetermined region; BjM, also an integer between 0 and 2, indicates the number of bases j in M1iM2i; and j represents A, T, G or C. Based on the occurrence probability Pj of base j and the number of reads of Aj, the cumulative probability P(F1iF2iM1iM2i) of each maternal and fetal genotype combination was determined as follows:(Eq. 3)

P(F1iF2iM1iM2i)=∑PjAj/∑Aj (3)

where Aj stands for the read count for base j (A, T, G or C). Based on the P(F1iF2iM1iM2i) of each maternal and fetal genotype combination, Pfinal (F1iF2iM1iM2i) was computed according to the formula (Eq. 4)

Pfinal (F1iF2i M1iM2i)= P(F1iF2iM1iM2i)/∑(P(F1iF2i M1iM2i)) (4)

The highest probability for the genotype combination was taken to be the final cumulative probability for that combination and was used in the Bayesian model. The predicted genotype at each locus was defined as the one with the highest posterior probability (patent PIDC3194001P).

In this way, the Bayesian model relied on parental variants and cfDNA preprocessing data as inputs, and it returned 10 posterior probabilities for 10 predicted fetal genotypes. Low-quality variants were eliminated from the Bayesian analysis (Supplementary Method S1).

### Prediction of Fetal Genotype Using a Haplotype-Based Method

We used a haplotype-based method based on sequential probability ratio testing (SPRT) and the “closest-variant” algorithm to predict fetal genotypes at AAAB and ABAB loci. First, we used longhap software (https://github.com/stLFR/stLFR_LongHap) to perform genome phasing based on the alignment and variant results of parental stLFR sequencing. Second, we deduced the inheritance of maternal haplotype at ABAA loci used a previous method (Lo et al., 2010). The SPRT was performed to determine whether the cumulative allele counts for SNVs along a haplotype block reached sufficient statistical confidence for Hap I or Hap II to be scored. SNVs for which statistical confidence was too low for a genotype call were considered “unclassified”. The maternally contributed alleles for these unclassified variants were inferred using the closest-variant algorithm 1, which predicted maternal or paternal inheritance. based on the inferred inheritance of the nearest variant within 200 kb in the same haplotype block. If the upstream and downstream variants showed different inherited haplotypes within a 200-kb region, these unclassified variants were not analyzed (Supplementary Figure S1). The closest-variant algorithm 1 was defined to predict maternal or paternal inheritance based on the inferred inheritance of the nearest variant within 200 kb in the same haplotype block. We defined the closest variant algorithm 2 to infer the paternally/maternally contributed allele for the variant using the inferred inheritance of the nearest variant within 500 Kb region of the same haplotype block (Supplementary Figure S1).

We defined the closest variant algorithm 2 to infer the paternally/maternally contributed allele for the variant using the inferred inheritance of the nearest variant within 500 Kb region of the same haplotype block (Supplementary Figure S1). In the case of InDels, we used the closest-variant algorithm 2 to infer paternal inheritance at AAAB and ABAB loci, and SPRT to predict maternal inheritance. The closest-variant algorithm 2 was also used to infer the maternally contributed alleles of unclassified variants. Low-confidence variants were filtered out in the SPRT (Supplementary Method S2).

### Combination of Bayesian- and Haplotype-Based Prediction of Fetal Genotype

We used the Bayesian model to infer paternally inherited alleles at AAAB loci, while we used the haplotype-based method to infer maternally inherited alleles at ABAA loci. In the case of ABAB loci, we first used the closest-variant algorithm 1 to determine the paternally inherited alleles based on the inferred inheritance at the closest AAAB locus determined by the Bayesian model within 200 kb in the same haplotype block, after which we conducted SPRT to predict maternally inherited alleles. The closest-variant algorithm 1 was used to determine maternal alleles of unclassified variants. Finally, we used the Bayesian model to predict fetal genotype at the remaining ABAA and ABAB loci for which the haplotype-based method did not predict genotype (Figure 2).

## Results

### Bayesian Model for Inferring Fetal Genotype

**FIGURE 1:**
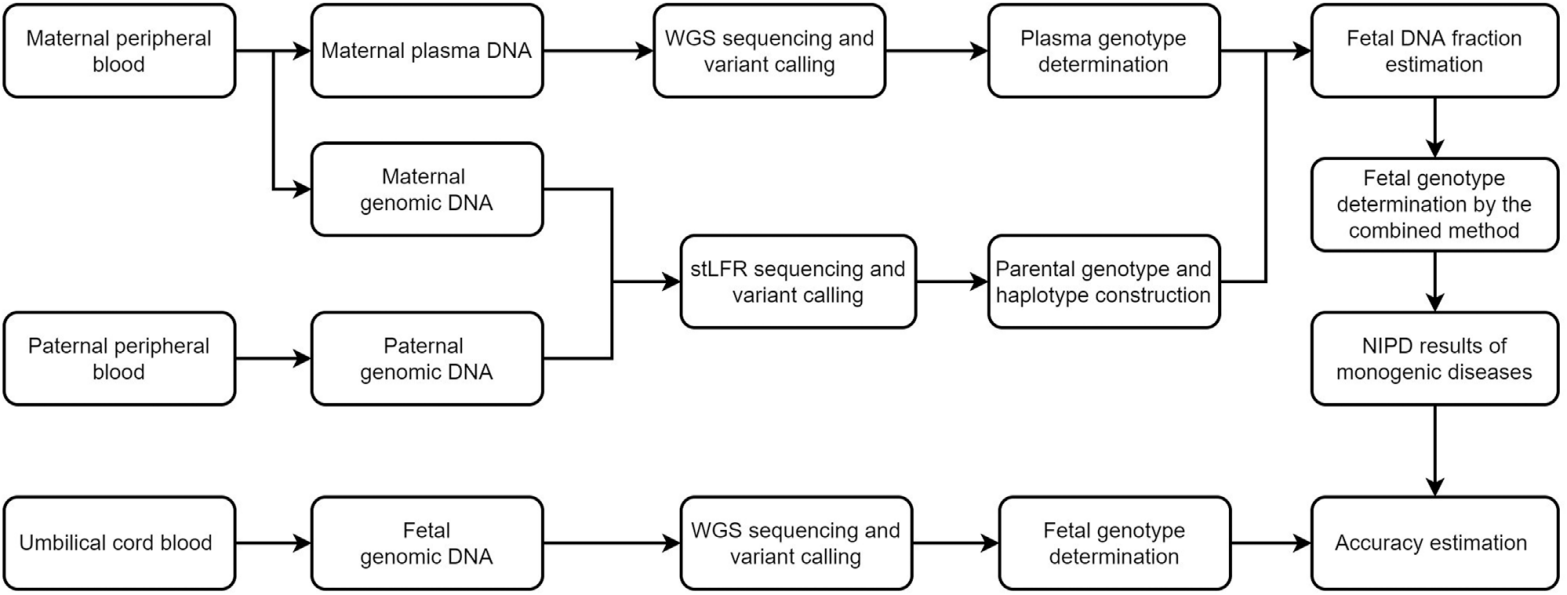
Schematic of this study. We first recruited five families and performed stLFR sequencing of parental genomic DNA and genome sequencing of cell-free DNA in maternal plasma. The fetal genome was successfully inferred using a combination of Bayesian- and haplotype-based prediction. Genome sequencing of fetal DNA in umbilical cord blood was used to determine the accuracy of our genotype inferences. WGS, whole genome sequencing, NIPD, non-invasive prenatal diagnosis; stLFR, single-tube long fragment reads.

Plasma cfDNA and genomic DNA from five healthy pregnant women and their husbands were sequenced. The women were aged 30.72 years (range, 28.5–32.6 years) bearing fetuses at a mean gestational age of 24.2 weeks (range, 13–33 weeks) (Supplementary Table S1). The cfDNA was sequenced at a depth of 100X (range, 112.03–256.12X); the genomic DNA, to a depth >30X (range, 28.77–68.39X; Supplementary Table S2, Figure 1). Umbilical cord blood DNA was sequenced at a depth of 48X (range, 41.85–52.34X; Supplementary Table S2). The estimated FF had a mean of 13% (range, 4%–27%; Supplementary Table S1).

**FIGURE 2:**
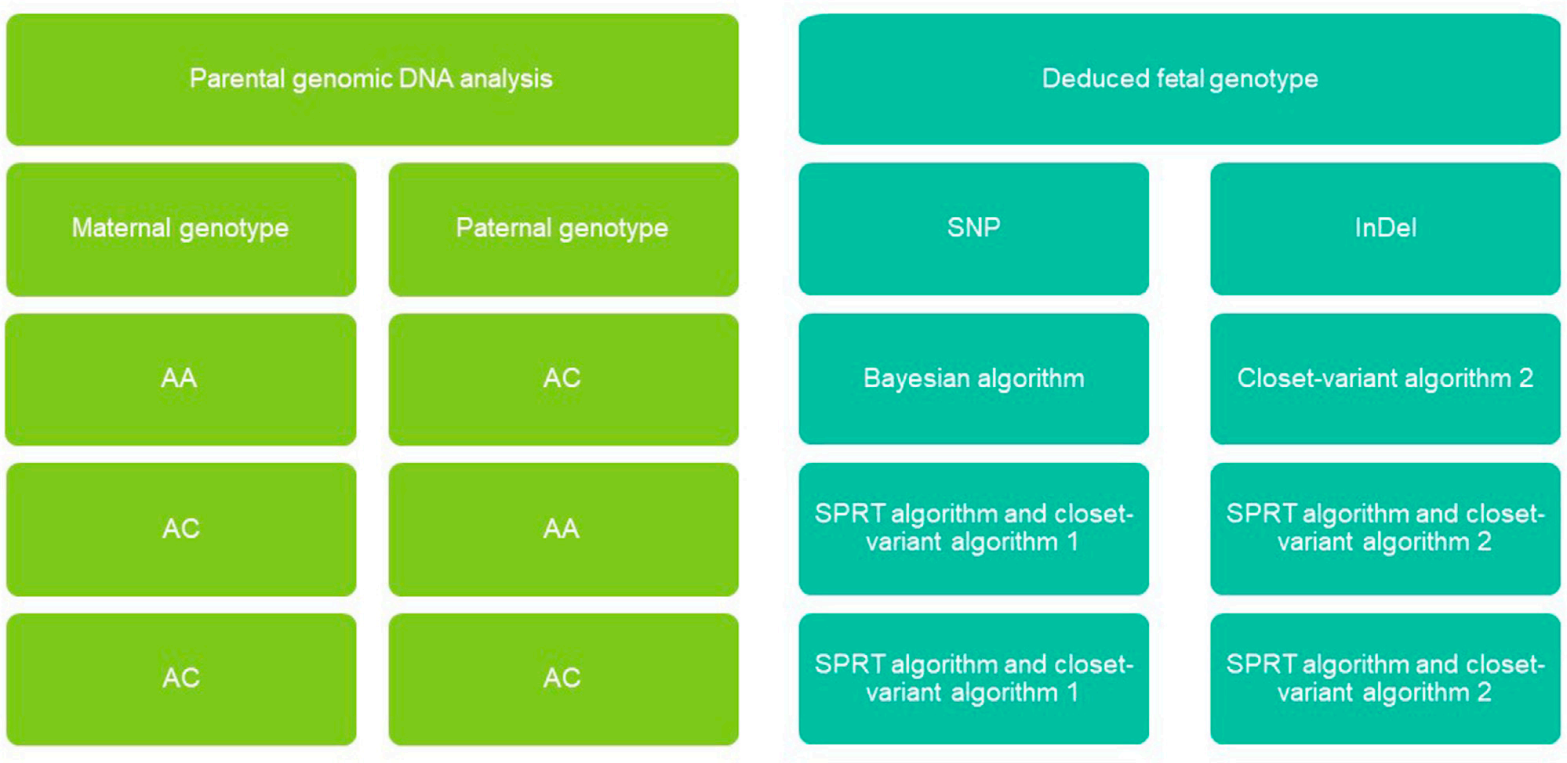
Non-invasive fetal genomic analysis based on cell-free DNA in maternal plasma. Parental combinations of single-nucleotide polymorphisms (SNVs) and insertions-deletions (InDel) were grouped into four types, each of which we predicted using a different strategy (see Methods). AA, homozygous; AB, heterozygous; SPRT, sequential probability ratio testing.

A Bayesian model to infer fetal SNVs showed the greatest accuracy with fetus JK-16, who had the highest FF (27%), and lowest accuracy with JK-53, who had the lowest FF (4%). These results indicate the strong influence of FF on fetal genotype inference. Increasing the cfDNA sequencing depth from 128 to 256.12X increased the accuracy of JK-53 genotyping (Table 1). The genotyping accuracy across the five families was 96.2 ± 5.8% at homozygous maternal and heterozygous paternal loci (AAAB), 74.6 ± 9.5% at ABAA loci, and 64.3 ± 11.9% at ABAB loci (Table 1; Figure 2).

**TABLE 1:** Performance metrics for inferring fetal SNPs in 5 healthy families.

Family	Heterozygous	The bayesian model		The haplotype-based method		The combined method	
		Accuracy (%)	Number of true predictions/number of loci	Accuracy (%)	Number of true predictions/number of loci	Accuracy (%)	Number of true predictions/number of loci
JK-7	AAAB loci	96.8	631788/652391				
	ABAA loci	72.6	909990/1253908	98.8	1171962/1186610	96.1	1209126/1253908
	ABAB loci	61.4	429479/698962	96.4	572296/593618	89.8	627424/698962
JK-16	AAAB loci	97.7	576306/590073				
	ABAA loci	91.8	1014224/1104405	99.2	1061980/1070070	98	1082868/1104405
	ABAB loci	85.9	540677/629324	97.5	562304/576574	94.9	597082/629324
JK-18	AAAB loci	96.4	463476/480708				
	ABAA loci	75.7	901292/1190790	99	1112107/1123516	96.7	1151107/1190790
	ABAB loci	65.9	439900/667062	92.7	488305/526841	86.5	576938/667062
JK-28	AAAB loci	95.2	313500/329369				
	ABAA loci	69.5	440760/633774	98.5	581675/590448	96	608483/633774
	ABAB loci	57.3	222910/388712	93.1	268695/288587	83.2	323556/388712
JK-53	AAAB loci	95.6	540046/564870				
(128X)	ABAA loci	59.6	667583/1120831	97.3	926919/952660	92.0	998262/1085313
	ABAB loci	49.4	325416/658784	89.1	363047/407606	74.7	481240/644645
JK-53	AAAB loci	95	590368/621392				
(256.12X)	ABAA loci	63.5	706502/1112899	97.7	979699/1002260	93.6	1041875/1112899
	ABAB loci	51	341350/668854	93.9	462865/492889	81.8	547115/668854

### Improving the Accuracy of Fetal Genotype Inference Using Haplotyping

**FIGURE 3:**
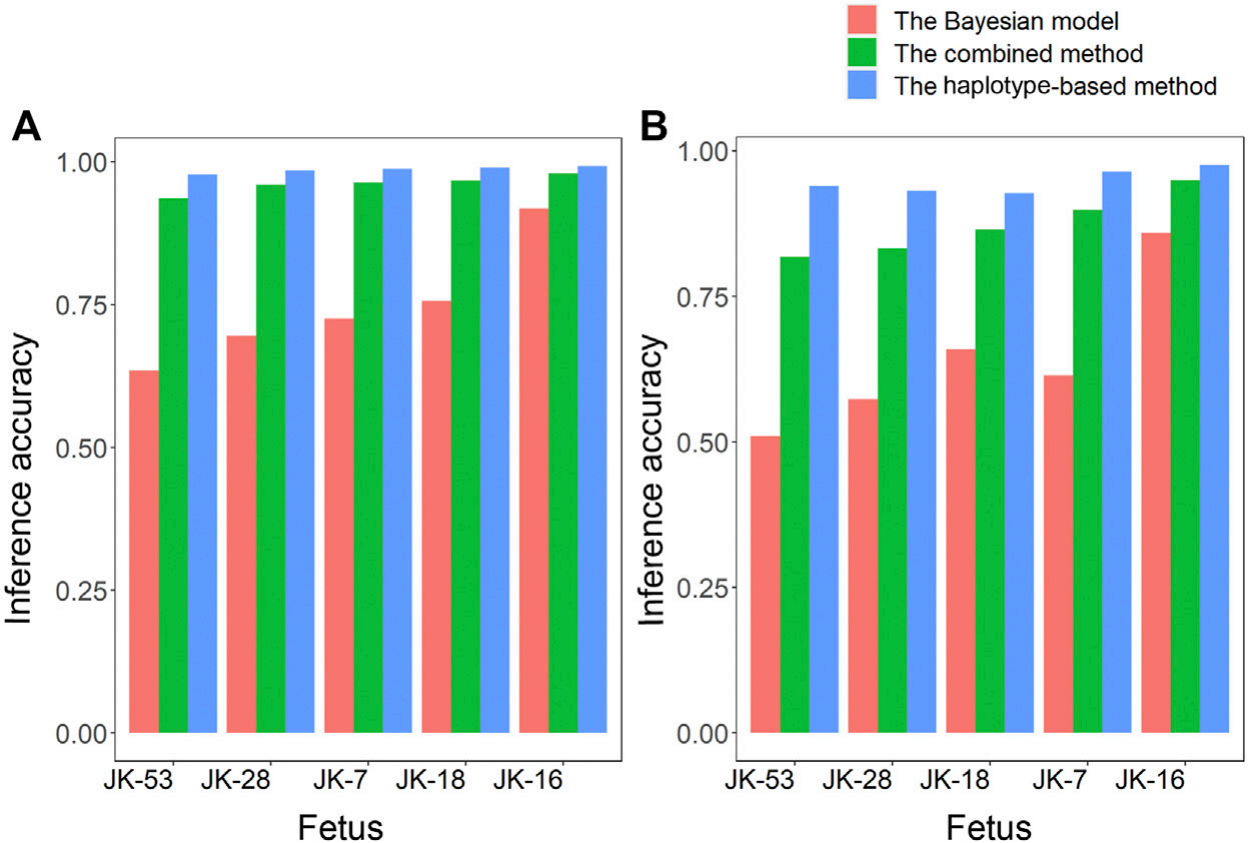
Comparison of how accurately fetal genotypes were inferred using the Bayesian model alone, the haplotype-based method alone, or the two methods together for **(A)** ABAA and **(B)** ABAB loci.

Given the Bayesian model’s poor performance at predicting fetal genotypes at ABAA and ABAB loci, we genotyped complex haploid subsets of maternal and paternal genomic DNA at these loci while preserving long-range contiguity (Figure 2). We directly phased over 99% of ABAA loci into long haplotype blocks, giving an average N50 of 18.72 Mb, and over 99% of ABAB loci into long haplotype blocks, giving an average N50 of 13.57 Mb (Supplementary Table S2). This haplotype-based method successfully classified 90%–97% of maternally inherited SNVs at ABAA loci and correctly predicted 98%–99% of SNVs (Table 1). Nevertheless, this haplotype-based method was unable to infer genotype at 3%–10% of ABAA loci, so we inferred these gaps using the Bayesian model. This combination of haplotype-based and Bayesian-based prediction (hereinafter referred to the combined method) gave SNV genotyping accuracies of 94%–98% at ABAA loci (Table 1; Figure 3A). The haplotype-based method successfully classified 74%–92% of maternally inherited SNVs at ABAB loci and correctly predicted 93%–98% of SNVs. Adding Bayesian inference to fill in gaps led to accuracies of 82%–95% (Table 1; Figure 3B).

We also utilized the haplotype-based method to infer paternal and maternal inheritances for fetal InDels, which was successful for 95% of all InDels (range, 93.6%–97%) across the fetuses. Accuracy was highest at ABAA loci (94.6%), followed by AAAB loci (80.2%) and ABAB loci (79.3%) (Supplementary Table S3, Supplementary Figure S2).

### Combining Haplotype- and Bayesian-Based Prediction for NIPD of Monogenic Diseases

We sequenced the cfDNA from the plasma of five pregnant women with a mean age of 29 years (range, 25–34 years) at a mean gestational age of 13 weeks (range, 11–18 weeks) whose fetuses were at risk for monogenic diseases. Mean FF was 0.15 (range, 0.08–0.20; Table 2). The cfDNA was sequenced at a depth of 121X (Supplementary Table S4). In parallel, stLFR sequencing of parental genomic DNA was performed at a depth of 24X. The combination of haplotype- and Bayesian-based prediction identified 8 pathogenic variants in five genes in the plasma cfDNA from all five mothers (Table 2, Supplementary Table S5). Our method correctly identified 6 heterozygous carriers of monogenic disease variants, including tetrahydrobiopterin deficiency hyperphenylalafivemia, Duchenne/Becker muscular dystrophy, ocular albinism, muscular dystrophy polysaccharide glycosylation deficiency A11 and deafness and 1 wildtype variant in a fetus at risk of Tetrahydrobiopterin deficiency hyperphenylalaninemia. The method also correctly predicted the heterozygous deletion c.8371delC (CDH23 in NM_022124) in a fetus at risk of deafness. The inferred fetal variants were validated by Sanger sequencing of DNA from umbilical cord blood, which revealed only one incorrect inference (Table 2, Supplementary Table S5). Therefore, in this sample of five fetuses, our method was able to non-invasively determine pathogenic variants with an accuracy of 87.5%.

**TABLE 2:** Summary of non-invasive prenatal diagnosis in 5 families with monogenic diseases by the combined model.

Family ID	Age	Gestational week	FF (%)	Monogenic diseases	Maternal genotype	Paternal genotype	Inferred fetal genotype (bayesian model prediction/haplotype-based prediction)
SFY-10	25	12	16	Tetrahydrobiopterin deficiency hyperphenylalaninemia	PTS(NM_000317) heterozygous c.73C > G	PTS(NM_000317) heterozygous c.155A > G	C/C (C/C, NA)
							A/G (A/G, A/G)
SFY-15	34	11	16	Duchenne/Becker Muscular Dystrophy	DMD (NM_004006) heterozygous c.187–2A > T	Wild-type	A/T (A/T, NA)
SFY-32	29	11	8	Deafness	CDH23(NM_022124) heterozygous c.8371delC	CDH23(NM_022124) heterozygous c.1606C > T	AC/A (NA, AC/A)
							C/T (C/T, NA)
SFY-05	27	18	20	Muscular dystrophy polysaccharide glycosylation deficiency A11	B3GALNT2(NM_152490) heterozygous c.181C > T	B3GALNT2(NM_152490) heterozygous c.261–2A > G	C/C (C/C, not in block)^a^
							A/G (A/G, NA)
SFY-18	30	13	14	Ocular albinism	GPR143 heterozygous c.885 + 748G > A	Wild-type	G/A (G/A, G/A)

a Indicates incorrect prediction.

## Discussion

In this study, we developed a Bayesian model for non-invasively inferring fetal genotypes based on sequencing of cfDNA in maternal plasma and of parental genomic DNA. The model accurately predicted fetal genotype at AAAB loci but poorly at ABAA and ABAB loci. By combining this approach with haplotype information, we accurately predicted SNVs and InDels at AAAB, ABAA and ABAB loci with high prediction accuracy despite a relatively low FF. We demonstrated the potential of our combined method for NIPD of monogenic diseases.

Over the past decade, several haplotype-based strategies have been reported for inferring fetal genotypes based on deep sequencing of maternal plasma (Lo et al., 2010; Fan et al., 2012b; Kitzman et al., 2012; Chen et al., 2013; Chan et al., 2016). In these strategies, the parental haplotype is determined directly (Kitzman et al., 2011; Lam et al., 2012) or derived from analysis of pedigrees (Lo et al., 2010; Chen et al., 2013; New et al., 2014) or founder haplotypes in selected populations (Zeevi et al., 2015). Haplotype blocks in these strategies average in size from 300 kb to >1 Mb, which restricts the resolution at which maternal inheritance of the fetus can be inferred (Lo et al., 2010; Fan et al., 2012b; Kitzman et al., 2012). The lack of complete haplotype information or genome phasing information in these strategies means that only ∼70% of paternally inherited haplotypes or ABAB loci can be analyzed (Fan et al., 2012b; Kitzman et al., 2012). A Bayesian method has been reported that can predict SNVs and InDels independently of the inheritance model and parental origin, but it cannot detect DNVs, multi-allelic loci or X-linked inheritance (Rabinowitz et al., 2019).

Our haplotype-based method was able to infer genotypes at AAAB, ABAA and ABAB loci with high accuracy, yet it could not do so for 3%–10% of ABAA loci or 8%–26% of ABAB loci. Therefore, we used a Bayesian model to predict the loci missed by the haplotype-based method. This combined approach allowed the accurate prediction of SNVs and InDels at all fetal loci at single-base resolution. Our method appears to be able to infer fetal genotype with much higher resolution than previously reported methods. For example, one previous method predicted only a fraction of ABAA loci, whereas it was unable to analyze ABAB loci (Chan et al., 2016). Another method predicted SNVs at ABAA loci with an accuracy of only 64.4%, but it was able to analyze SNVs at only some ABAB loci for lack of paternal haplotype information (Fan et al., 2012b). In contrast, our method correctly predicted SNVs at AAAB, ABAA and ABAB loci with accuracies of 82%–95%. In addition, our method accurately predicted SNVs and InDels. For example, our method predicted Indels at AAAB, ABAA and ABAB loci with accuracies of 79%–95%. In fact, during analysis of five fetuses at risk of monogenic disease, our method detected five disease-causing mutations. Thus, our method appears to be the only one reported so far that can comprehensively predict SNVs and InDels. Moreover, our method delivered accurate predictions at FFs as low as 4%, much lower than in previously published methods (Kitzman et al., 2012; Rabinowitz et al., 2019), indicating the potential for NIPD early in pregnancy.

By using stLFR technology we were able to determine parental haplotypes without the need for proband DNA and with a much shallower sequencing depth than a previously published method (Chan et al., 2016). Our approach may become accessible to more institutions as genome-wide direct phasing becomes less expensive and technically demanding (Che et al., 2020). At the same time, our method needs to be improved to increase its clinical feasibility, such as increasing the accuracy of predicting SNVs at ABAB loci and InDels at AAAB and ABAB loci. The Bayesian model that we applied here calculates the likelihood of the fetal genotype using the maternal genotype and FF. Adding other features to the model may improve its ability to discriminate fetal and maternal reads; such features may include fragment size (Rabinowitz et al., 2019) and clusters of preferred ending positions of fetal fragments (Chan et al., 2016). Another approach to improve inference accuracy may be to apply scalable FF amplification technology (Welker et al., 2020).

## Conclusion

We have established a haplotype- and Bayesian-based method that can accurately predict fetal genotype at single-base resolution. Our method may be useful for accurately recovering fetal genomes and for NIPD of monogenic diseases caused by SNVs or InDels.

## Data Availability

The data reported in this study are available from the CNGB Sequence Archive in the CNGBdb database under accession number CNP0001437.
