# Supplementary material for: Non-Invasive Prenatal Diagnosis of Monogenic Disorders Through Bayesian- and Haplotype-Based Prediction of Fetal Genotype
Source: Front Genet. 2022 Jul 1;13:911369. doi: 10.3389/fgene.2022.911369 (PMC9283829; doi:10.3389/fgene.2022.911369)
Supplement: Supplementary file 1 [file DataSheet1.docx]

Supplementary method1. The criteria to remove low quality variants in the Bayesian model analysis.

The following variants were eliminated from the Bayesian model analysis: (1) loci with read depth < 20 in maternal plasma or read depth < 15 in parental samples; (2) low quality parental or fetal variants which met any of the following criteria: QualByDepth (QD) < 2.0, RMSMappingQuality (MQ) < 50.0, FisherStrand (FS) > 60.0, StrandOddsRatio (SOR) > 3.0, MappingQualityRankSumTest (MQRankSum) < -12.5, ReadPosRankSumTest (ReadPosRankSum) < -8.0; (3) heterozygous variants with fraction of alterative allele reads smaller than 0.2 or greater than 0.8.

Supplementary method2. The criteria to remove low confidence variants in the SPRT analysis.

The variants which met the following criteria were eliminated from the SPRT analysis: (1) loci with read depth < 20 in maternal plasma or read depth < 15 in parental samples; (2) heterozygous variants with fraction of alterative allele reads smaller than 0.2 or greater than 0.8; (3) variants without parental haplotype information.

Supplementary Table1. Clinical characteristics of the 5 healthy families

| Family ID | Age of mother | Age of father | Height of mother | Weight of mother | Gestational weeks | NT(mm) | NIPT result for T21, T18 and T13 | Birth method | Fetal gender | Fetal fraction | Maternal complications |
| --- | --- | --- | --- | --- | --- | --- | --- | --- | --- | --- | --- |
| JK-7 | 31.6 | 35.3 | 168 | 60 | 26w+5 | 1.5 | Negative | Vaginal delivery | Female | 0.09 | No |
| JK-16 | 32.6 | 31.8 | 159 | 48 | 13w | 1.9 | Negative | Vaginal delivery | Female | 0.27 | No |
| JK-18 | 28.5 | 29.4 | 175 | 66 | 33w+6 | 1.6 | Negative | Vaginal delivery | Female | 0.14 | No |
| JK-28 | 30.7 | 31.0 | 164 | 53 | 29w+6 | 2.3 | Negative | Vaginal delivery | Female | 0.12 | No |
| JK-53 | 30.2 | 32.5 | 162 | 47 | 20w+2 | 2.7 | Negative | Vaginal delivery | Female | 0.04 | Intrahepatic cholestasis |

Supplementary Table2. summary of sequencing data in the 5 healthy families

| Family ID | Sequencing method | Number of raw reads | Number of mapped reads | Mapping rate | Average depth | Coverage | Duplicate Rate | N50 | Fraction of phased SNPs |
| --- | --- | --- | --- | --- | --- | --- | --- | --- | --- |
| JK-7 | WGS on cord blood cells | 1233682194 | 1231279450 | 99.81% | 41.85 | 99.16% | 3.75% |  |  |
| JK-7 | WGS on maternal cfDNA | 4930998263 | 4899353438 | 99.36% | 167.1 | 99.27% | 32.58% |  |  |
| JK-7 | StLFR on maternal blood cells | 2246895792 | 2213288894 | 98.50% | 68.39 | 99.32% | 53.12% | 38692373 | 99.8% |
| JK-7 | StLFR on paternal blood cells | 1847735151 | 1825890887 | 98.82% | 57.29 | 99.87% | 46.48% | 13595319 | 99.88% |
| JK-16 | WGS on cord blood cells | 1547702979 | 1544031563 | 99.76% | 52.34 | 99.16% | 3.15% |  |  |
| JK-16 | WGS on maternal cfDNA | 4470433381 | 4427313912 | 99.04% | 150.99 | 99.27% | 31.17% |  |  |
| JK-16 | StLFR on maternal blood cells | 1126578266 | 1093285410 | 97.04% | 34.05 | 99.20% | 37.45% | 43167036 | 99.78% |
| JK-16 | StLFR on paternal blood cells | 1071372133 | 1036265674 | 96.72% | 32.43 | 99.81% | 36.28% | 43812809 | 99.84% |
| JK-18 | WGS on cord blood cells | 1393244829 | 1391262033 | 99.86% | 47.27 | 99.14% | 3.49% |  |  |
| JK-18 | WGS on maternal cfDNA | 3410469123 | 3388616846 | 99.36% | 115.68 | 99.26% | 22.39% |  |  |
| JK-18 | StLFR on maternal blood cells | 1437373627 | 1404317757 | 97.70% | 42.84 | 99.27% | 36.91% | 7009682 | 99.76% |
| JK-18 | StLFR on paternal blood cells | 1419559729 | 1363089772 | 96.02% | 40.42 | 99.88% | 43.98% | 4303673 | 99.72% |
| JK-28 | WGS on cord blood cells | 1388613164 | 1386761047 | 99.87% | 47.38 | 99.13% | 4.18% |  |  |
| JK-28 | WGS on maternal cfDNA | 3311517554 | 3286161812 | 99.23% | 112.03 | 99.27% | 21.11% |  |  |
| JK-28 | StLFR on maternal blood cells | 1444610729 | 1412560944 | 97.78% | 42.08 | 99.27% | 38.33% | 1178766 | 99.05% |
| JK-28 | StLFR on paternal blood cells | 1284525331 | 1246303246 | 97.02% | 37.82 | 99.85% | 42.77% | 1424125 | 99.26% |
| JK-53 | Cord blood cell WGS | 1480341904 | 1477844272 | 99.83% | 50.33 | 99.13% | 4.23% |  |  |
| JK-53 | Maternal plasma cfDNA | 8909756646 | 8870299941 | 99.56% | 256.12 | 99.29% | 40.84% |  |  |
| JK-53 | Maternal blood cell stLFR | 933259760 | 919002827 | 98.47% | 28.77 | 99.12% | 17.24% | 3552388 | 99.66% |
| JK-53 | Paternal blood cell stLFR | 1055522782 | 1035088382 | 98.06% | 31.53 | 99.76% | 13.31% | 4708438 | 99.64% |

Supplementary Table3. Performance metrics for inferring fetal indels in 5 healthy families

|  | Number of true predictions/Total number of AAAB loci | AAAB  Accuracy | Number of true predictions/Total number of ABAA loci | ABAA  Accuracy | Number of true predictions/Total number of ABAB loci | ABAB  Accuracy | FF | Total number of indels | Indels with SPRT results | Fraction of indels with SPRT results |
| --- | --- | --- | --- | --- | --- | --- | --- | --- | --- | --- |
| JK-7 | 95446/123935 | 77% | 190093/199209 | 95.4% | 95069/123443 | 77% | 0.09 | 460212 | 446587 | 97% |
| JK-16 | 73714/95314 | 77.3% | 142989/147016 | 97.3% | 74754/93231 | 80.2% | 0.27 | 358513 | 335561 | 93.6% |
| JK-18 | 82721/104098 | 79.5% | 158053/170435 | 92.7% | 84550/106000 | 79.8% | 0.14 | 402516 | 380533 | 94.5% |
| JK-28 | 37904/42794 | 88.6% | 71967/75357 | 95.5% | 40697/48082 | 84.6% | 0.12 | 174955 | 166233 | 95% |
| JK-53 | 70971/90470 | 78.4% | 131633/142619 | 92.3% | 69269/92726 | 74.7% | 0.04 | 342825 | 325815 | 95% |

Supplementary table4. summary of sequencing data in the 9 families with monogenic diseases

| Family ID | Sequencing method | Number of raw reads | Number of mapped reads | Mapping rate | Average depth | Coverage | Duplicate Rate |  | N50 | Fraction of phased SNPs |
| --- | --- | --- | --- | --- | --- | --- | --- | --- | --- | --- |
| SFY-05 | WGS on maternal cfDNA | 4302980383 | 4297898766 | 99.88% | 117.52 | 99.18% | 19.71% |  |  |  |
| SFY-05 | WGS on cord blood cells | 1353352923 | 1351467907 | 99.86% | 44.58 | 99.14% | 1.44% |  |  |  |
| SFY-05 | StLFR on maternal blood cells | 1243175414 | 1228180488 | 98.79% | 20.31 | 99.10% | 46.44% |  | 1507317 | 97.93% |
| SFY-05 | StLFR on paternal blood cells | 1207496158 | 1195440285 | 99.00% | 32.63 | 99.76% | 15.34% |  | 2280192 | 98.85% |
| SFY-10 | WGS on maternal cfDNA | 5065896724 | 5059689894 | 99.88% | 130.79 | 99.80% | 24.09% |  |  |  |
| SFY-10 | WGS on cord blood cells | 1213447293 | 1211423622 | 99.83% | 40.37 | 99.81% | 1.12% |  |  |  |
| SFY-10 | StLFR on maternal blood cells | 816654203 | 807762191 | 98.91% | 22.53 | 99.03% | 12.49% |  | 1440301 | 98.35% |
| SFY-10 | StLFR on paternal blood cells | 484961614 | 482423939 | 99.48% | 14.33 | 99.60% | 11.36% |  | 1079654 | 98.23% |
| SFY-15 | WGS on maternal cfDNA | 5101582918 | 5094457150 | 99.86% | 113.83 | 99.86% | 34.25% |  |  |  |
| SFY-15 | WGS on cord blood cells | 1684086799 | 1681731316 | 99.86% | 55.79 | 99.86% | 1.32% |  |  |  |
| SFY-15 | StLFR on maternal blood cells | 673554987 | 670968051 | 99.62% | 16.17 | 98.83% | 27.43% |  | 22580522 | 98.63% |
| SFY-15 | StLFR on paternal blood cells | 420181906 | 417253926 | 99.30% | 10.35 | 99.38% | 24.81% |  | 7265439 | 98.29% |
| SFY-18 | WGS on maternal cfDNA | 3776205123 | 3767335423 | 99.77% | 104 | 99.85% | 18.62% |  |  |  |
| SFY-18 | WGS on cord blood cells | 1601882647 | 1600110673 | 99.89% | 50.49 | 99.82% | 4.46% |  |  |  |
| SFY-18 | StLFR on maternal blood cells | 882445457 | 879815219 | 99.70% | 23.61 | 98.98% | 19.66% |  | 24819614 | 98.86% |
| SFY-18 | StLFR on paternal blood cells | 1424061086 | 1410535191 | 99.05% | 39.67 | 99.16% | 13.94% |  | 5307728 | 98.54% |
| SFY-32 | WGS on maternal cfDNA | 4113891426 | 4098674631 | 99.63% | 116.65 | 99.24% | 16.71% |  |  |  |
| SFY-32 | WGS on cord blood cells | 1011943863 | 1010639051 | 99.87% | 33.73 | 99.07% | 1.86% |  |  |  |
| SFY-32 | StLFR on maternal blood cells | 899185808 | 894716576 | 99.50% | 17.03 | 98.93% | 43.16% |  | 3049785 | 99.19% |
| SFY-32 | StLFR on paternal blood cells | 482277796 | 480838573 | 99.70% | 11.87 | 99.27% | 25.05% |  | 1567691 | 98.87% |

Supplementary table5. Summary of non-invasive prenatal diagnosis in 9 families with monogenic diseases by the combined model

| Family ID | Types of inheritance | Monogenic diseases | Inferred fetal genotype (Bayesian model prediction/haplotype-based prediction) | Sanger sequencing validation |  |
| --- | --- | --- | --- | --- | --- |
| SFY-10 | AR | Tetrahydrobiopterin deficiency hyperphenylalaninemia | C/C (C/C, NA)  A/G (A/G, A/G) | Wildtype  PTS(NM_000317) heterozygous c.155A>G |  |
| SFY-15 | XL | Duchenne/Becker Muscular Dystrophy | A/T (A/T, NA) | DMD(NM_004006) heterozygous c.187-2A>T |  |
| SFY-32 | AR | Deafness | AC/A(NA, AC/A)  C/T(C/T, NA) | CDH23(NM_022124) heterozygous c.8371delC  CDH23(NM_022124) heterozygous c.1606C>T |  |
| SFY-05 | AR | Muscular dystrophy polysaccharide glycosylation deficiency A11 | C/C (C/C, not in block) *  A/G (A/G, NA) | B3GALNT2(NM_152490) heterozygous c.181C>T*  B3GALNT2(NM_152490) heterozygous c.261-2A>G |  |
|  |  |  |  |  |  |
| SFY-18 | XL | Ocular albinism | G/A (G/A, G/A) | GPR143 heterozygous c.885+748G>A |  |

Notably, AD, AR and XL refer to autosomal dominant, autosomal recessive, X-linked inheritance patterns respectively. * indicates incorrect prediction validated by sanger sequencing.


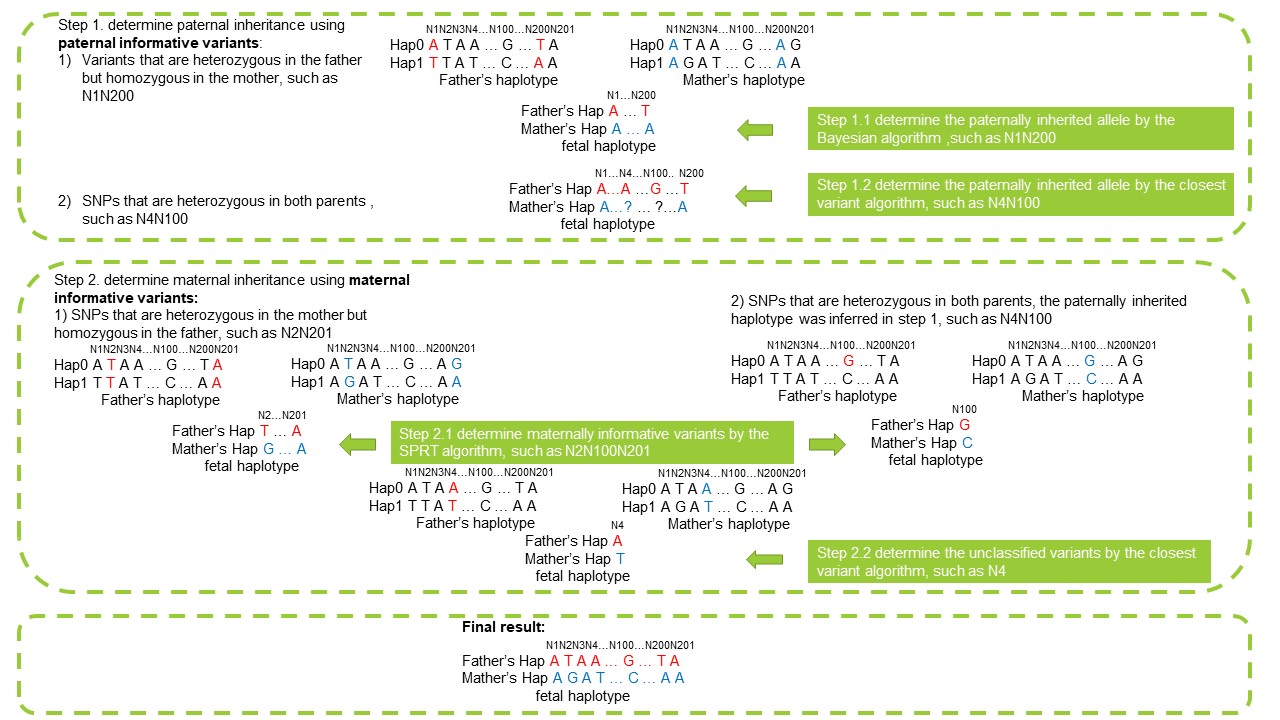


Supplementary Figure1. The Noninvasive fetal genomic analysis from maternal plasma DNA and parental genomic DNA. We firstly used the Bayesian model to infer paternally inherited alleles at paternal-specific sites. The closest variant algorithm was utilized to determine the paternally inherited alleles using the inferred inheritance of the closest “paternal-only” heterozygous site determined by the Bayesian model within 100kb region of the same haplotype block at shared heterozygous sites. Then the SPRT method to implemented to infer maternally inherited alleles at ABAA and ABAB sites. The maternal inheritance of unclassified variants was determined by the closest variant algorithm.


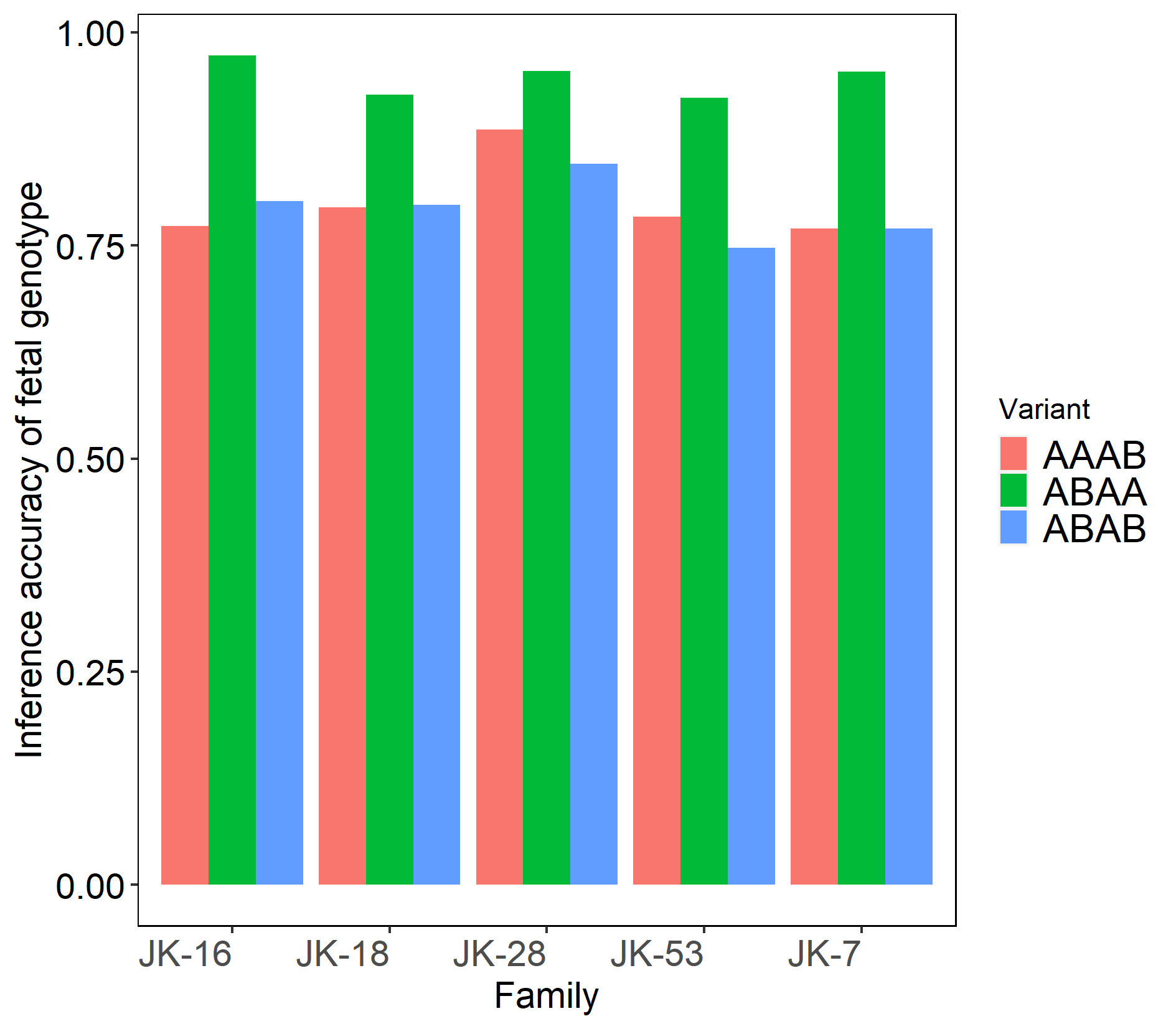


Supplementary Figure2. The comparison of inference accuracy of fetal genotypes at AAAB, ABAA and ABAB loci of indels.
